# Structural basis for transthyretin amyloid formation in vitreous body of the eye

**DOI:** 10.1038/s41467-021-27481-4

**Published:** 2021-12-08

**Authors:** Irina Iakovleva, Michael Hall, Melanie Oelker, Linda Sandblad, Intissar Anan, A. Elisabeth Sauer-Eriksson

**Affiliations:** 1 Department of Chemistry, Umeå University, SE-901 87 Umeå, Sweden; 2 Department of Public Health and Clinical Medicine, Umeå University, SE-901 87 Umeå, Sweden; 3 Wallenberg Centre for Molecular Medicine, Umeå University, Umeå, Sweden

**Keywords:** Cryoelectron microscopy, Hereditary eye disease, Cryoelectron microscopy, Diseases, Structural biology

## Abstract

Amyloid transthyretin (ATTR) amyloidosis is characterized by the abnormal accumulation of ATTR fibrils in multiple organs. However, the structure of ATTR fibrils from the eye is poorly understood. Here, we used cryo-EM to structurally characterize vitreous body ATTR fibrils. These structures were distinct from previously characterized heart fibrils, even though both have the same mutation and type A pathology. Differences were observed at several structural levels: in both the number and arrangement of protofilaments, and the conformation of the protein fibril in each layer of protofilaments. Thus, our results show that ATTR protein structure and its assembly into protofilaments in the type A fibrils can vary between patients carrying the same mutation. By analyzing and matching the interfaces between the amino acids in the ATTR fibril with those in the natively folded TTR, we are able to propose a mechanism for the structural conversion of TTR into a fibrillar form.

## Abstract (web summary)

Systemic ATTR amyloidosis causes the abnormal accumulation of ATTR fibrils formed from the human plasma protein transthyretin (TTR) in multiple organs including the eye. Here, the authors present a 3.2 Å cryo-EM structure of an ATTR fibril isolated from the vitreous body of an ATTR patient’s eye and discuss the mechanism for the structural conversion of TTR into a fibrillar form.

## Introduction

Amyloids are highly ordered protein self-assemblies of β-sheet-rich structures with characteristic cross-β conformations that aggregate as straight, unbranched fibrils 100–200 Å in diameter. Accumulation of amyloid in various tissues is linked to several fatal disorders known as amyloidoses. The human plasma protein transthyretin (TTR) is one of more than 30 proteins known to cause amyloidosis^1–3^. TTR is mainly produced in the liver (90%)^4^, and it serves as a transporter of thyroxine hormone and vitamin A^5,6^. Extracellular deposition of amyloid TTR (ATTR) is associated with diseases referred to as ATTR amyloidoses. Hereditary ATTR (ATTRv) amyloidosis is a pathology caused by single-point mutations in the TTR gene, and currently more than a hundred pathogenic single-point variants are known^7^. Of these, the substitution of valine for methionine at position 30 (Val30Met) is the most common variant associated with ATTRv^8^. The clinical manifestation of ATTRv is peripheral polyneuropathy affecting motor, sensory, and autonomic functions, with the accumulation of amyloid deposits mainly along the peripheral nerves and in the kidney, spleen, eye, and heart^4^. Ocular involvement in ATTRv includes the deposition of amyloid in the vitreous body of the eye (the gel-like substance between the lens and retina of the eyeball), and this condition affects up to 35% of patients with ATTRv^9^.

Two forms of fibril composition exist in patients with ATTR amyloidosis. Type A fibrils contain a high proportion of fragmented ATTR that are not found in type B fibrils, which are composed almost entirely of the full-length protein. Furthermore, type A fibrils are short and thin while type B fibrils are long and thick^10^. The type of fibril in Val30Met patients influences the phenotype of the disease and the selection of therapy^11^. Patients with type A fibrils have a late onset of the disease and typically develop cardiomyopathy. Their prognosis is worse^12^ than for patients with type B fibrils who have early-onset disease but with less myocardial involvement^13^. For patients with ATTRv Val30Met amyloidosis, type A fibril composition is very common and might even be the general rule^14^.

TTR fibrillogenesis follows a downhill polymerization mechanism^15–17^. In its native functional state, TTR forms a homotetramer (a dimer of dimers), with a central channel containing a thyroid-hormone binding site on either side^18,19^. Each subunit comprises an eight-stranded β-barrel with one short alpha helix. Dissociation of the tetramer into monomers is the rate-limiting step in TTR fibrillogenesis, and once the monomers partially unfold the polymerization cascade is initiated^15–17^. For monomer misfolding, the instability of the CD-loop, i.e., the short loop connecting β-strands C and D, plays an essential role^20–23^. Several studies show that amino acid substitutions in this region increase the rate of TTR fibrillation^21,24^. Furthermore, the CD-loop is prone to partial proteolysis that yields potently fibrillogenic N- and C-terminal fragments^25,26^. The main proteolytic cleavage site is between residues Lys48 and Thr49, but additional cleavage sites exist within the residue range Ala45–Glu54^14,25,27^. However, it is an ongoing debate whether the TTR proteolysis occurs before or after fibril incorporation^10,25^.

Recently, a high-resolution structure of amyloid fibrils from the heart of a patient with ATTRv Val30Met amyloidosis provided the first insights of how natively folded TTR protein changes its shape to become incorporated into a single amyloid protofilament^26^. In that structure, as well as in other amyloid ones^28–30^, the proteins or peptides forming the amyloid fibrils reorganize as flat discs that stack perfectly onto each other to form a protofilament. These stacks are stabilized by hydrogen bonds between parallel β-strands organized in the characteristic cross-β-strand conformation^31–33^ common to all amyloid fibrils^28–30^.

Polymorphism is a common, if not conserved, feature of amyloid fibrils in vivo and describes the variations in fibril morphological structures formed by identical protein chains^34^. Less is known how polymorphism occurs and differs between fibrils formed in different organs. Considerable research has been devoted toward studying cardiac ATTR depositions, demonstrating the polymorphism of ATTR amyloid fibrils in the heart^10,26,35^. ATTR amyloid fibrils are also found in the vitreous body of the eye. The vitreous amyloidosis caused by these amyloid fibrils is a condition found exclusively in ATTRv^36^. Interestingly, it has been postulated that vitreous ATTR has its own local source of TTR synthesis—the retinal pigment epithelium—which implies that vitreous amyloidosis may have different misfolding and aggregation pathways^37,38^. Thus, it is warranted to characterize ATTR fibrils in the eye for comparison with fibrils from other organs. Here, we structurally characterized vitreous body fibrils from a patient with ATTRv Val30Met amyloidosis and showed that they are polymorphic and mainly consist of multi-protofilaments. Moreover, we determined the 3.2 Å cryo-EM structure of the fibril consisting of two protofilaments and compared it to the single protofilament fibril structure from the heart^26^. Our results show that ATTR protein can adopt different folds and arrangements into protofilaments of type A fibrils. Finally, by comparing the fibrillar structure of ATTR with the structure of soluble TTR, we are able to propose a mechanism for how TTR unfolds and misfolds in the formation of amyloid protofilaments.

## Results

### Polymorphism of ATTR fibrils in the vitreous body of the eye

Vitreous body material was collected by vitrectomy from the eye of a Swedish carrier of the ATTR Val30Met variant who suffered from both polyneuropathy and cardiomyopathy. The diagnostic pathology identified type A fibrillogenesis. The vitreous body of the eye contains relatively few proteins^39^. This simplifies the purification of amyloid, and the ATTR fibrils could be isolated by centrifugation. Biochemical analysis showed a mixture of the Val30Met and wild-type TTR proteins and confirmed the presence of one predominant Thr49-Glu127 fragment (Supplementary Figs. 1 and 2), in agreement with previous investigations on vitreous body fibrils^38,40,41^.

**Fig. 1:**
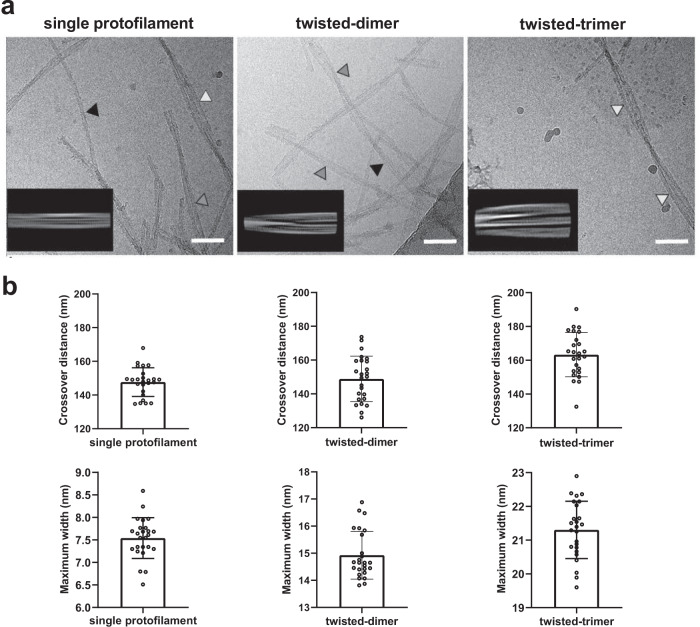
Cryo-EM images and analysis of ATTR fibrils from the vitreous body. **a** Representative cryo-EM micrographs: single protofilament, black arrowhead; twisted-dimer, gray arrowhead; twisted-trimer, white arrowhead. The 2D class averages of each filament type are shown as insets. Scale bars, 50 nm. **b** Estimated crossover distances and maximum widths of ATTR fibrils in the micrographs. Box plots show the mean values ± standard deviations from *n* = 25 independent measurements for each type of fibril. The cross-distances are 147.7 ± 8.5 nm for monomer, 148.9 ± 13.4 nm for twisted-dimer, and 163.3 ± 13.1 nm for twisted-trimer. The maximum width for monomer is 7.5 ± 0.5 nm, for twisted-dimer is 14.9 ± 0.9 nm and 21.3 ± 0.8 nm for twisted-trimer. The maximum width and helical periodicity were analyzed by Fiji (ImageJ) 1.52 and GraphPad Prism 9.2.0 softwares. Source data for (**b**) are provided in a Source data file.

Analysis of the vitreous body material by cryo-EM revealed a broad range of ATTR fibril morphologies, from which we could clearly differentiate three distinct types of fibrils: single protofilaments (~23%); two intertwined protofilaments (termed twisted-dimer; ~62%); and three intertwined protofilaments (termed twisted-trimer; ~13%) (Fig. 1a). In addition, we observed other types of fibrils consisting of multiple (up to five) intertwined protofilaments (2%) as well as the coexistence of twisted-dimers and twisted-trimers in a single fibril (Supplementary Fig. 3). Of these polymorphs, only twisted-dimers were present in sufficient amounts for high-resolution cryo-EM structural analysis.

We characterized the different fibril morphologies on the cryo-EM grid by measuring the crossover distance and the maximum width of the single protofilament, twisted-dimer, and twisted-trimer. The estimated crossover distances showed a high degree of heterogeneity also within each polymorph. For monomers, crossover distances ranged between 130 and 160 nm, for the twisted-dimers they were 130–170 nm, and for the twisted-trimers they were 140–190 nm (Fig. 1b). The width of the fibrils correlated with their number of protofilaments. The fibrils consisting of a single protofilament had a width of ~7.5 nm, and the width of the twisted-dimer was approximately the width of two single protofilaments. Likewise, the twisted-trimer had a width approximately the size of three protofilaments lying side-by-side (Fig. 1b). These results suggest that maturation of vitreous amyloid fibrils is achieved by side-by-side intertwining of additional protofilaments for each polymorph.

### Cryo-EM structure and the architecture of twisted-dimer ATTR fibrils

**Fig. 2:**
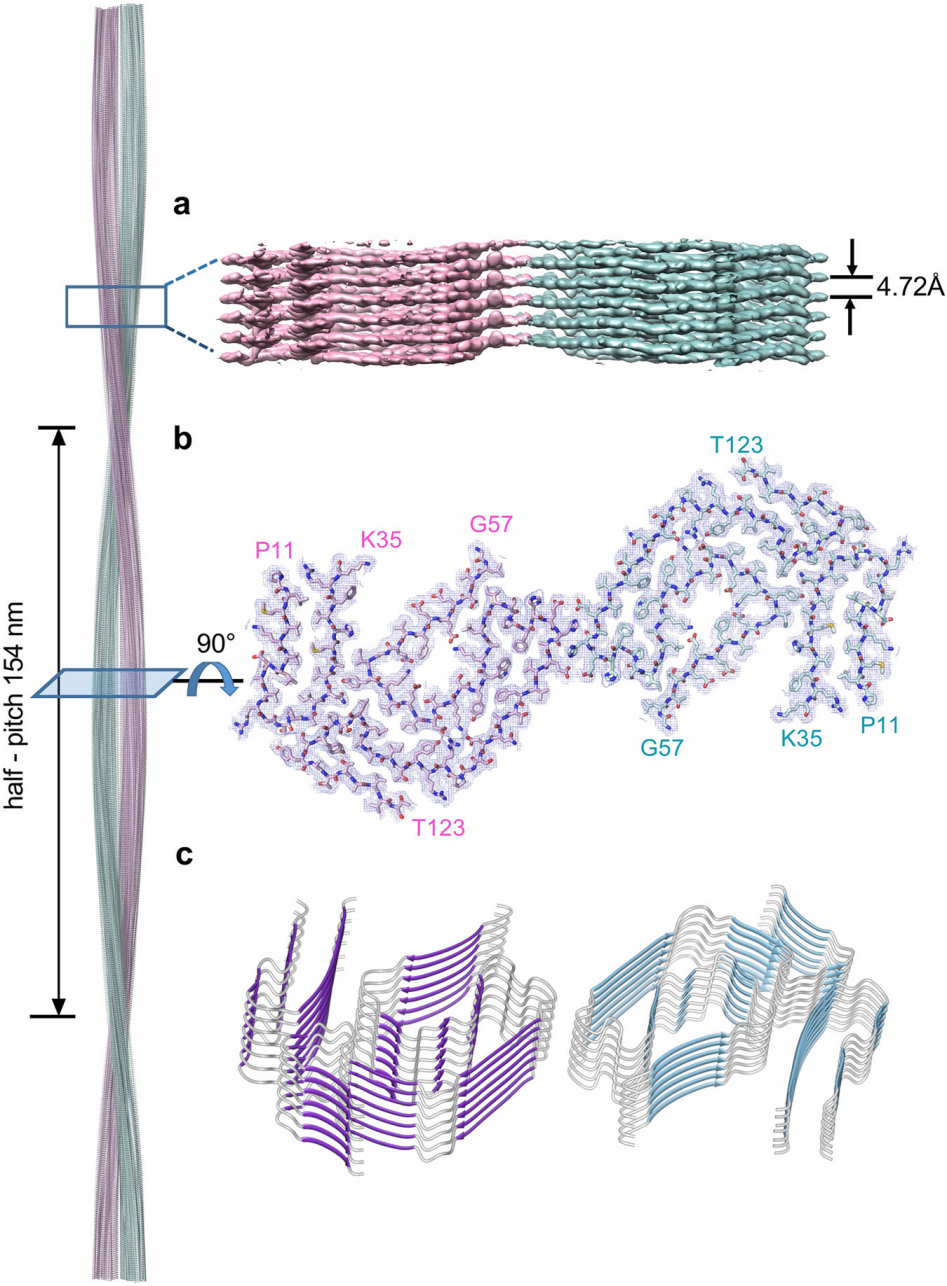
Cryo-EM reconstruction of the vitreous body ATTR twisted-dimer. **a** Side view of the reconstructed 3D map with the indicated half-pitch and helical rise. The two protofilaments in the twisted-dimer are colored cyan and pink, respectively. **b** Cross-section view of the density map (blue) with the modeled structure. Residues in the two protofilaments are colored in pink and cyan, respectively. **c** Ribbon drawing of seven layers of the twisted-dimer stack.

Using cryo-EM we obtained the structure of the vitreous body ATTR Val30Met twisted-dimer consisting of two intertwined protofilaments. The model was built in the cryo-EM map at a spatial resolution of 3.2 Å (for statistics see Supplementary Tables 1 and 2 and Supplementary Fig. 4). The ATTR fibril proteins in each protofilament had identical conformations related by a twofold symmetry axis (C2 symmetry) along the fibril long axis. Consequently, layers of fibril proteins within one protofilament stacked in register with layers within the other protofilament (Fig. 2a). This conformation is consistent with other amyloid fibrils built from multiple protofilaments^42,43^. The protofilaments intertwined along the C2-axis into a left-handed helix with a helical crossover distance of 154 nm, a helical twist of –0.55°, and a helical rise of 4.72 Å, as determined by the layer line from the power spectrum from reference-free 2D class averages (Supplementary Fig. 5). The continuous Coulomb potential regions in each protofilament were modeled with two fragments of ATTR—the N-terminal fragment comprising residues Pro11–Lys35 and the C-terminal fragment comprising residues Gly57–Thr123 (Fig. 2b, c). No well-defined density was observed for residues Ala36–His56.

### Rearrangement of secondary structures in TTR is required for ATTR fibril formation

**Fig. 3:**
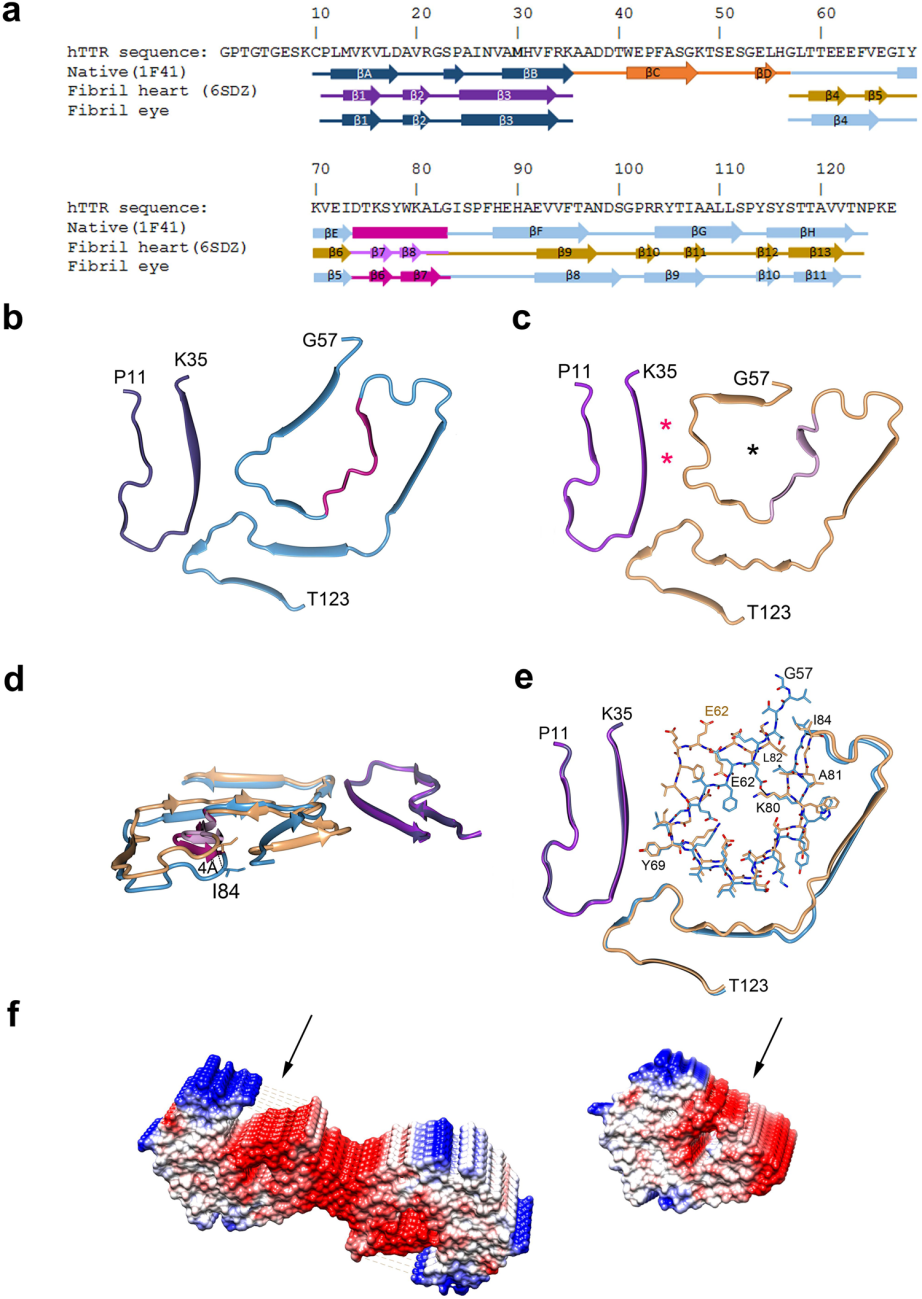
Location of secondary structures in the vitreous body and heart ATTR fibrils. **a** Primary sequence of TTR Val30Met with secondary structure elements indicated for natively folded TTR, heart ATTR fibrils, and vitreous ATTR fibrils. **b** Cα trace of one protofilament of vitreous ATTR twisted-dimer fibril protein. The secondary structures in **b**–**e** are color-coded as in **a**. **c** Cα trace of the protofilament of heart ATTR fibril protein (PDB-ID 6SDZ). The black asterisk marks an internal cavity not present in the vitreous fibril protein. The red asterisks mark two densities not present in vitreous ATTR. **d** Superposition of the vitreous and heart fibril protein structures shown in **b** and **c**. **e** Structural changes in the Gly57–Ile84 region. In particular, Ala81–Leu82 have swapped their positions. **f** Electrostatic surface potentials of vitreous twisted-dimers (left) and heart fibrils (right). The black arrows indicate identical positions on the two fibrils with markedly different surfaces and electrostatic potentials due to the conformational changes shown in (**d**, **e**).

In its soluble form, TTR exists as a homotetramer in which the 127 amino acids in each monomer fold into a β-barrel structure formed by two β-sheets (β-strands D–A-G-H and β-strands C-B-E-F). One short α-helical region connects β-strands βE and βF (Supplementary Fig. 6)^19^. In contrast, each fibril protein in the twisted-dimer has 11 β-strands (strands β1–β11). The positions of these β-strands do not correspond to those of the β-strands in the natively folded protein (Fig. 3a). In particular, two additional shorter β-strands (β6 and β7) are formed in the twisted-dimer fibril protein as a result of rearrangement of the sole α-helix in the structure of TTR. These structural changes convert the 3D barrel structure into an almost flat disc-like one. The discs are packed into stacks, i.e., protofilaments. Stabilizing interactions between the discs in one stack include main-chain hydrogen bonds between the parallel β-strands, side-chain contacts including aromatic stacking, and crossover salt bridges and hydrogen bonds (Supplementary Fig. 7).

### Vitreous body and heart muscle ATTR Val30Met protofilaments are structurally not identical

We compared the structure of the vitreous body twisted-dimer with the recent cryo-EM structure of an ATTR Val30Met fibril isolated from the heart^26^. The heart fibril structure was determined from a single protofilament, whereas we characterized the two-protofilament fibril, i.e., the twisted-dimer. The individual vitreous body and heart fibril proteins in their respective discs were made up of N- and C-terminal ATTR fragments consisting of residues Pro11–Lys35 and Gly57–Thr123 (Fig. 3b, c). The β-strands in the vitreous body fibril protein, however, were more extended and fewer in number, with β-strands β4 and β5 and β-strands β10 and β11 in the heart fibril protein being the equivalent to β-strands β4 and β9, respectively, in the vitreous body fibril protein (Fig. 3a). In total, the vitreous body fibril protein comprised 11 β-strands, whereas the fibril protein of the heart muscle contained 13.

Superimposition of the fibril proteins of the vitreous body and heart showed that some, but not all, regions could be aligned. Superimposition of residues Pro11–Lys35, i.e., the N-terminal fragment, gave a root-mean-square distance of 0.5 Å for the corresponding main-chain atoms. With the N-terminal region superimposed, residues Ile68–Lys76 and Ala108–Thr123 also superimposed well. Residues Ser77–Ile107 were structurally identical in both fibril proteins but did not run at the same level. These residues in the vitreous body fibril proteins ran almost in the same plane as the N-terminal fragment, but they rose above the plane in the heart fibril protein by as much as 4 Å, as measured between the Cα atoms of Ile84 (Fig. 3d).

However, the major structural difference between the two fibril proteins was the folding of the first 11 residues of the C-terminal fragment, residues Gly57–Ile68. In vitreous body fibril protein, these residues form a long β-strand placed diagonally over the core of the fibril protein. In the heart fibril protein, the same residues form two short β-strands placed in an L-shaped structure. This fold creates an internal cavity that is missing in the vitreous body fibril protein (Fig. 3c)^26^. Nor are the conformations of residues Tyr78–Leu82 the same. In particular, Ala81 and Leu82 have different positions. Together these structural changes create different packing interactions between residues Lys80–Ile84 and Leu58–Glu62 (Fig. 3e). Independent of the fold, the faces of both fibril proteins are highly hydrophobic, which contributes to the high affinity of the protein subunits for stacking on top of each other. However, the different conformations of the proteins greatly affected the charge distribution and accessibility of the exposed surface in the resulting protofilaments (Fig. 3f).

### Inter-protofilament interactions

**Fig. 4:**
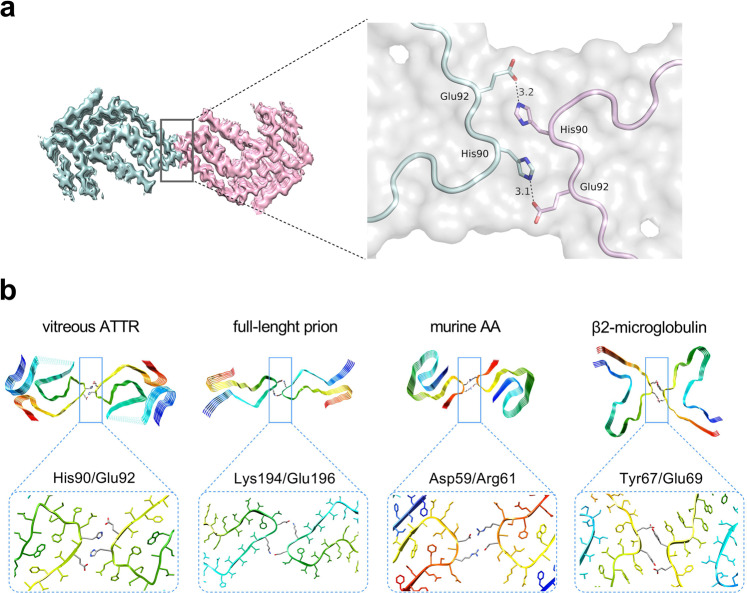
Inter-protofilament interactions. **a** The interface in the twisted-dimer of ATTR vitreous body fibrils. **b** Comparison of the interfaces between protofilaments in four amyloid fibrils—vitreous body ATTR fibril, full-length human prion (PDB-ID 6LNI), murine amyloid A (AA) (PDB-ID 6DSO), and β2-microglobulin (PDB-ID 6GK3).

The protofilaments in the twisted-dimer are held together by crossover hydrogen bonds. The side-chain imidazole ring of His90 from one protofilament hydrogen bonds to the side-chain carboxyl group of Glu92 of the other protofilament (Fig. 4a). With a pKa of ~6.0, a fraction (~15–25%) of the imidazole rings have a positive charge at pH 7.0–7.2, which aids in packing interactions at the interface. By comparing our twisted-dimer structure with other high-resolution structures of amyloid fibrils^43–45^, we identified three amyloid fibrils that form inter-protofilaments interactions similar to ours despite the lack of sequence similarity (Fig. 4b). None of these three structures involved histidine side chains, although all of the fibrils formed inter-protofilament contacts at sharp turns in the protein chain and involved two residues and their symmetry-related counterparts. There was a preference to form electrostatic interactions, in the form of salt bridges or hydrogen bonds, to stabilize the interactions at the relatively small surface area interface (Fig. 4). Other known inter-protofilament contacts based on hydrophobic residues form steric zipper-type interactions, for example, Tau^28^, α-synuclein^29^, and Aβ(1–42)^30^.

## Discussion

Unlike visceral ATTR deposits, the amyloid in the eye derives from TTR locally synthesized by the retina^37,38^. Therefore, the ATTR protein produced in the eye may form a structure distinct from other affected organs. In this study, we used cryo-EM to structurally study ATTR fibrils isolated from the vitreous body of the eye. The analysis revealed multiple fibril morphologies, demonstrating the polymorphism of ATTR fibrils in the eye. Morphological differences were perpetuated at several structural levels, indicating that the polymorphs differ in terms of the number of protofilaments and the periodicity of the helical twist of the fibrils. Interestingly, the protofilaments in the multi-protofilament fibrils packed side-by-side to form flat and ribbon-like fibrils (“planar packing”). This suggests that maturation of vitreous ATTR into multi-protofilament fibrils occurs, not by forming bundles of protofilaments, but by a previously unobserved side-by-side intertwining of the protofilaments (Fig. 1 and Supplementary Fig. 3).

The overall morphology of vitreous amyloid fibrils identified here is uncharacteristic for a type A fibril composition. They are relatively long and vary in the number of protofilaments attached. This contrasts with type A morphology of fibrils isolated from the cardiac^10,26^ and peripheral nerve systems^46^, where they are relatively short with a width corresponding to the size of the single protofilament. Thus, our results show that type A fibrils are polymorphic and vary in the number of protofilaments.

The reason behind the formation of various polymorphs in the eye and other organs mentioned above is currently unclear. This may be due to the local TTR synthesis in the eye and modifications of the ATTR protein, e.g., proteolysis induced by the different environments. However, earlier results suggest that amyloid fibrils in systemic amyloidosis share a common fibril structure and that their formation follows the same rules regardless of the organ in which they originate^47^. Therefore, there may also be underlying patient-specific factors, meaning that fibril morphologies can differ between individuals with the same mutation.

Most of the fibrils (~80%) in the vitreous amyloid sample contain multiple protofilaments, thus assembly into higher-order structures seems favorable for this fibril protein. Our comparison of the cryo-EM structure of the vitreous body twisted-dimer, with that of the heart single protofilament fibril^26^, highlights similarities as well as distinctions. Both fibril models include N- and C-terminal fragments of identical length, but which differ in the packing of the fragments in their respective protofilaments (Fig. 3). In particular, the conformation of the first 11 residues of the C-terminal fragment differ from each other. This structural difference is manifested in a displacement of the C-terminal fragment by up to 4 Å in the vicinity of the inter-protofilament boundary in the twisted-dimer (residues His90–Glu92). The structural variations of the fibril proteins directly affect the surface properties of the resulting fibrils, as well as their accessibility (Fig. 3f), which may explain why multi-protofilament fibrils were not observed to a larger extent in the heart tissue^26^.

Proteolytic processing plays an essential role in ATTR fibrillogenesis and yields C-terminal fragments that differ in length in various organs. Interestingly, our results as well as previous studies have shown that vitreous amyloid fibrils are predominantly cleaved between residues Lys48 and Thr49^38,40,41^ (Supplementary Figs. 1 and 2), whereas fibrils from cardiac tissue are cleaved at multiple sites within the residue range Ala45–Glu54^26,38^. This implies that the fraction of Thr49-Glu127 fragments could be higher in vitreous fibrils than in heart fibrils. In a low-pass filtered map, we observed densities not present in the heart structure, in which we could extend our model of the C-terminal fragment to include two conformations of residues Thr49–His56. In the more well-defined density, the modeled residues reach towards the site of inter-protofilament interactions (Supplementary Fig. 8). Although these residues could be only partially modeled, their presence in the vitreous fibrils suggests that C-terminal fragments of varying lengths pack differently in the layer structures, which might explain the high fraction of twisted-dimers in our vitreous sample.

Non-protein factors present in the extracellular matrix assist folding and assembly of amyloid proteins in vivo. Their distribution changes the microenvironments in which they occur, and affects the distribution, extent, and architecture of amyloid fibrils^48^. Non-protein densities have been observed in cryo-EM structures of ex vivo amyloid fibrils^26,42,49,50^. In tau filaments from the brain of individuals with chronic traumatic encephalopathy, this density has been interpreted as incorporation of an unidentified hydrophobic cofactor. Moreover, the presence of this density in a novel tau filament fold, points to a folding role of the bound cofactor^42^. Interestingly, the cryo-EM map of the heart fibril protein contains two extra spherical densities, positioned between the N- and C-terminal fragments (see Fig. 3c). These densities are relatively small and are not related to the density of the ATTR protein^26^. Side chains from residues Ala29, His31, Phe33, Val65, and Ile68 surround their positions, which indicates their hydrophobic nature. Due to their small size and hydrophobic environment, these densities in heart ATTR may correspond to small folding-assisting molecules as has been suggested^42^. Therefore, based on the different folds of the C-terminal fragments in the heart and vitreous fibril proteins and the absence of connecting densities in the structure of the latter, it is reasonable to assume that molecular inclusions may also contribute to the folding of ATTR protein and determine the overall structure of the fibril. However, the identity of these molecules and their role in the formation of the fibril structure remain to be established.

**Fig. 5:**
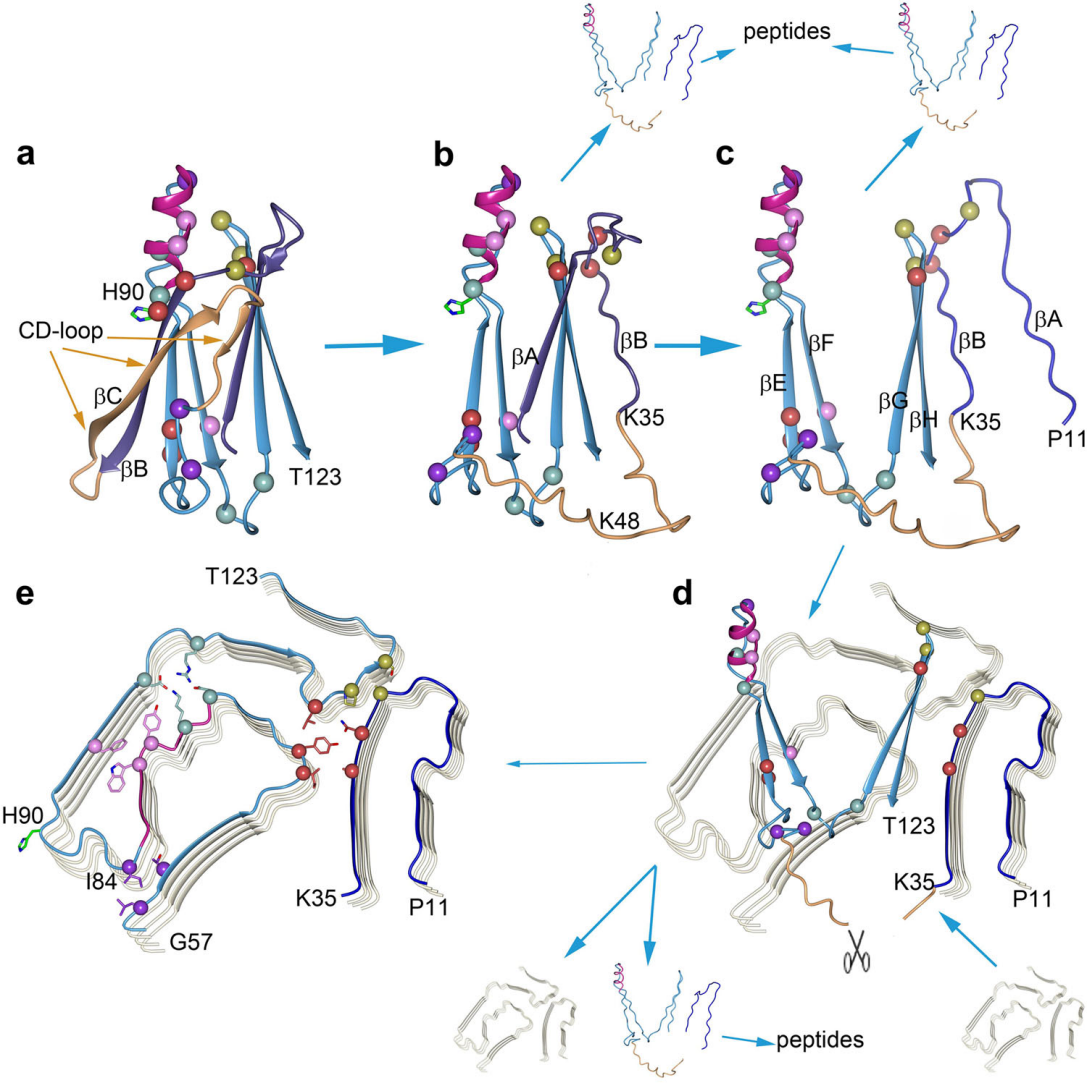
Possible molecular mechanism for ATTR formation. Refolding of the TTR protein into the fibril is likely aided by interactions with either another partly folded TTR molecule like the ones seen in (**b**, **c**) or with pre-formed fibrils like the one seen in (**d**). This way the TTR protein forms fibrils before reaching an entirely unfolded state. The natively folded monomer structure of TTR seen in (**a**) is color-coded according to Supplementary Fig. 6. Five contact interfaces in the protein fibril structure seen in (**e**) have their Cα atoms highlighted as colored spheres. The respective Cα atoms are also colored in the TTR structure. **a** Amyloid formation is initiated by the detachment of the CD-loop (orange). **b** Due to the strong interactions between βB and βC (11 main-chain hydrogen bonds), movements of the CD-loop occasionally also include βB, separating it from βE. **c** The upper part of the barrel now lacks crossover strands, which permits the barrel to open up, and strand βA to be released from strand βG. **d** Subsequently, structural changes occur that lead to the continuous transition of fibril formation and in no particular order, including proteolytic cleavage at position Lys48–Thr49 and further processing of the cleaved CD-loop. The positions of critical residues in the remaining two β-hairpins suggest that—facilitated by the presence of the fibril—the remainder of the barrel opens up and flattens out. **e** Fibril formation progresses only for a small fraction of misfolded TTR, in case productive β-strand–β-strand interactions between ATTR fibril proteins were able to form.

TTR proteins that are soluble under normal conditions must undergo substantial structural changes to form ATTR. TTR fibrillogenesis follows a downhill polymerization mechanism, in which dissociation of the TTR homotetramer and partial unfolding of the monomers initiate the amyloid aggregation cascade^15–17^. However, it is not known how the resulting monomers rearrange or how they become so perfectly incorporated into the amyloid structure. X-ray^51^ and NMR structures^23^, as well as mass spectrometry^52^ and solution scattering^53^, have identified non-native partially unfolded monomeric species. However, the cryo-EM ATTR fibril structures show that the structural conversion is far more extensive^26^, our work. Essentially, the 3D β-barrel structure of the natively folded monomer has to reorganize into a flat 2D-layer. Little is known about this process, but two properties are known to play a crucial pathogenic role in ATTR fibril formation. These are the flexibility of the CD-loop of the monomer and the proteolytic cleavage of the peptide bond between Lys48 and Thr49 in the same loop^20–23,54,55^. To learn more about the TTR fibrillization process, we analyzed the ATTR fibril protein and identified residues whose side chains made the crucial interactions that stabilized each layer of the ATTR protofilament. Then we compared the position of these with the corresponding ones in the natively folded monomer structure of soluble TTR. On the basis of this comparison, we propose a plausible mechanism for the unfolding and misfolding of TTR in the formation of fibrils (Fig. 5a). We find that monomers do not need to be largely unfolded to facilitate fibril formation, as has been suggested^26^. Rather, conditional unfolding is part of the fibril formation process, in which unfolded residues are stabilized by successive hydrogen bond formation to residues in the growing filament.

In this study, we showed that the ATTR protein can form different fibril morphologies even within the same type of pathology. The cryo-EM structures of ATTR fibrils isolated from the heart and eye show that the formation of different fibril morphologies depends on the conformation of the fibril proteins that constitute their building blocks. The reason for variation in fibril protein structure could be either patient-specific, or due to modifications induced by the local microenvironment of the organ in which the amyloid fibril is formed. These insights may be useful for the development of new therapies against ATTR amyloid.

## Methods

### Source and purification of fibrils

Vitreous material was collected by vitrectomy from the eye of a 72-year-old male Swedish carrier of the ATTR Val30Met variant who suffered from both polyneuropathy and cardiomyopathy. ATTR nature of amyloid was established in 2017. Previous biopsy analysis confirmed type A fibril composition and heterozygosity for Val30Met. The material collection and use were approved by the ethical review board of Umeå University, reference number 2018-329-32 M. Informed consent was obtained from the patient for the analysis of the amyloid deposits.

Fibrils were stored in 150 mM NaCl, 20 mM Tris -HCl pH 7.4 solution at room temperature. Fibrils from the vitreous humor were recovered by centrifugation for 1 min at 1000 × *g*. The supernatant was discarded and the fibril-containing pellet was washed three times with 20 mM Tris-HCl pH 7.4. After the final wash, the pellet was dissolved in water and immediately vitrified in liquid ethane for transmission electron microscopy.

### Denaturation protein gel electrophoresis

The fibrils were dissolved in 6 M urea, phosphate-buffered saline pH 7.4 solution. In total, 10 μL of the fibril-containing solution was mixed with 4 µL of 5x SDS loading buffer. The samples were boiled at 95 °C for 10 min in a heating block. The samples were loaded onto 12% Tricine-SDS-PAGE. Electrophoresis was carried out at room temperature (RT) applying a voltage of 130 V. The separated proteins were visualized with silver staining (Pierce™ Silver Stain Kit, Thermo ScientificTM).

### Mass spectrometry

TTR samples (0.2–0.5 mg/mL) in 40 mM sodium phosphate buffer, 6 M guanidine hydrochloride, at pH 6.8 were analyzed using automated sample preparation on a LEAP H/D-X PAL™ platform interfaced to an Ultimate 3000 micro-LC and Orbitrap Q Exactive Plus MS.

For pepsin digestion, samples of 5 μL were diluted with 45 μL, 0.2 M TCEP, 4 M urea, pH 2.5 and directly injected and subjected to online pepsin digestion at RT (in-house immobilized pepsin column, 2.1 × 30 mm). The online digestion and trapping were performed for 4 min using a flow of 50 µL/min of 0.1% formic acid (FA), pH 2.5. Peptides generated by pepsin digestion were subjected to online SPE on a PepMap300 C18 trap column (1 × 15 mm) and washed with 0.1% FA for 60 s. Thereafter, the trap column was switched in-line with a reversed-phase analytical column, Hypersil GOLD, particle size 1.9 µm, 1 × 50 mm. Separation was performed at 1 °C using a gradient of 5–50% B over 8 min and then from 50 to 90% B for 5 min, the mobile phases were 0.1% FA (A) and 95% acetonitrile/0.1% FA (B). Following the separation, the trap and column were equilibrated at 5% organic content, until the next injection. The needle port and sample loop were cleaned three times after each injection with mobile phase 5% MeOH/0.1% FA, followed by 90% MeOH/0.1% FA and a final wash of 5% MeOH/0.1% FA. In order to minimize carry-over, a full blank was run between each sample injection. Separated peptides were analyzed on a Q Exactive Plus MS, equipped with a HESI source operated at a capillary temperature of 275 °C, sheath gas 12, Aux gas 2 and sweep gas 1 (au). For the identification of generated peptides, spectra were acquired using data-dependent MS/MS (HCD).

For analysis of intact TTR, the same sample preparation protocol as for the digestion described above was used but by passing the online pepsin column, MS full scan spectra at a setting of 70 K resolution, AGC 3e6, max allowed injection time of 200 ms, and scan range 700–1800 *m/Z* were collected.

### Data analysis of mass spectrometry

PEAKS Studio X Bioinformatics Solutions Inc. (BSI, Waterloo, Canada) was used for peptide identification after pepsin digestion. The search was done using both the Uniprot (Human 2021_03) and a FASTA file with only the TTR (wt and V30M) sequence, search criteria was a mass error tolerance of 15 ppm and a fragment mass error tolerance of 0.05 Da, allowing for fully unspecific cleavage by pepsin. BioPharma Finder 3.2 was used to perform the intact protein deconvolution and targeted peptide mapping analyses.

### Cryo-EM

For cryo-EM, aliquots of 4.0 μM of purified ATTR fibrils were applied to glow-discharged holey carbon grids (Quantifoil Cu R1.2/1.3, 200 mesh), blotted with filter paper, and plunge-frozen in liquid ethane using an FEI Vitrobot Mark IV. The cryo-EM images were collected on a Thermo Fisher Titan Krios microscope operated at 300 kV at a magnification of ×130,000 (1.041 Å/pixel) equipped with a BioQuantum-filtered (20 eV slit width) Gatan K2 Summit detector. Images were collected as 20-frame movies for a total accumulated dose of 28.4 electrons per Å^2^. In total 17,863 images were recorded during two separate data collections using the EPU 2.7.0 software (Thermo Fisher) with defocus values ranging from –0.7 to –1.6 μm and in steps of 0.3 μm in both datasets.

### Helical reconstruction

The recorded frames were motion-corrected using MotionCor2 1.3.0^56^ implemented with dose weighting. Estimation of the contrast transfer function (CTF) for each non-dose-weighted micrograph was done using CTFFIND 4.1.8^57^, and all reconstructions were performed using the helical reconstruction methods in RELION 3.1^58,59^. Closer analysis of the micrographs using Fiji (ImageJ) 1.52 software indicated multiple fibril morphologies of varying diameters and crossover distances within the same sample. The filaments with two protein stacks (two protofilaments) and a crossover distance corresponding to the same distance were carefully selected and segmented into a small box of 220 pixels with an inter-box distance of 14.3 Å. A total of 130,212 segments were extracted from the two data sets and subjected to 2D classification. The 2D class averages showed a clear separation of beta-strands along the length of the fibril, and corresponding diffraction patterns in the power spectrum revealed a helical rise of ~4.72 Å. The helical twist of –0.57° was calculated from the estimated crossover distance of the filaments. An initial 3D model was generated from reference-free 2D class averages in the 256-pixel box by applying the *relion_helix_inimodel*2*d* program^59^ implemented in RELION 3.1. The 256-pixel box resulted in a greater number of 2D class averages and gave a better initial 3D model. Using the selected segments in the box of 220 pixels and the initial de novo model, we performed a 3D classification with optimization of the helical twist and rise. The segments contributing to the best class were selected for a subsequent 3D gold-standard auto-refinement. The resulting map showed incomplete separation of β-strands along the helical axis. Due to the twofold mirror symmetry in the reconstruction, we also explored the possibility of the fibrils having a translation component as part of their symmetry. C1 with pseudo-2_1_ screw symmetry and C2 symmetry were separately imposed in subsequent refinements. Application of the pseudo-2_1_ screw symmetry did not improve the quality of the map, and artifacts due to incorrect symmetry were evident. Refinement with C2 symmetry resulted in a model with complete separation of β-strands along the helical axis and higher resolution, so subsequent refinements used C2 symmetry. The final 3D auto-refinement of 27,778 selected segments converged onto a helical twist of –0.552°. The helical rise of 4.72 Å was kept fixed, and all refinements used a 30% value for the corresponding *helical_z_percentage* parameter. Following CTF refinement and Bayesian polishing, the final refinement yielded a model at 3.2 Å resolution. The final reconstruction was sharpened by applying the standard post-processing procedure in RELION, resulting in a B-factor of –73.12 Å^2^. Helical symmetry was imposed on the post-processed map using the relion_helix_toolbox program^58^.

### Model building and refinement

A single chain of the ATTR fibril structure from the heart (PDB-ID 6SDZ^26^) was used as the starting molecule and was docked in the map density for one protofilament and then manually rebuilt using Coot 0.8.9.2^60^ to fit into the cryo-EM map. A copy of this model was then placed in the second protofilament of the fibril, and a stack of seven subunits was generated and subjected to multiple rounds of real-space refinement with non-crystallographic symmetry restraints in PHENIX 1.17.1^61^. All refinements were performed using map information at 3.2 Å resolution. Structural validation was done with MolProbity^62^ as implemented in PHENIX 1.17.1. For final statistics of the refined model, see Supplementary Table 2.

### Protein structure representation

UCSF Chimera^63^ was used for creating the images of the density maps and protein models. The structure of the heart ATTR fibril protein shown in Fig. 3 was obtained from previously published coordinates in protein data bank (PDB) entry 6SDZ^26^. For the interface comparisons demonstrated in Fig. 4, the structures for the fibril proteins were obtained from the following published coordinates: 6GK3^43^, 6LNI^44^, and 6DSO^45^.

### Reporting summary

Further information on experimental design is available in the Nature Research Reporting Summary linked to this paper.

## Supplementary information

Supplementary Information

Reporting summary

## Data Availability

The structure of the twisted-dimer of ATTR Val30Met fibril from the vitreous body was deposited in the Electron Microscopy Data Bank with accession number EMD-12794. In addition, the atomic model built into the 3.2-Å structure was deposited in the Protein Data Bank with entry code: 7OB4. Other structural models used in this study are available in the Protein Data bank with entry codes: 6SDZ (amyloid transthyretin from the heart), 6LNI (full-length human prion), 6DSO (murine amyloid A), and 6GK3 (β2-microglobulin). Other data that support the findings of this study are available from the corresponding authors upon reasonable request. Source data are provided with this paper.

## Source data

Source Data
