# Supplementary material for: Structural basis for transthyretin amyloid formation in vitreous body of the eye
Source: Nat Commun. 2021 Dec 8;12:7141. doi: 10.1038/s41467-021-27481-4 (PMC8654999; doi:10.1038/s41467-021-27481-4)
Supplement: Supplementary file 1 — Supplementary Information [file 41467_2021_27481_MOESM1_ESM.pdf]

**Supplementary Information**

# Structural basis for transthyretin amyloid formation in vitreous body of the eye

I. Iakovleva et al.

**Supplementary Figure 1**

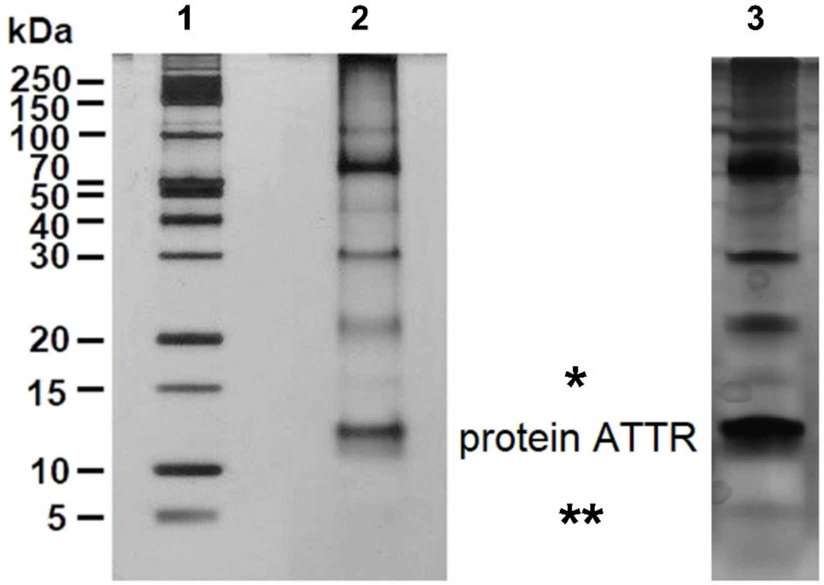


**Supplementary Figure 1**

**Gel electrophoresis of ATTR fibril sample.** Representative SDS-PAGE gel of urea solubilized ATTR fibrils visualized with silver staining. Line 1: protein ladder; line 2: vitreous fibrils sample; line 3: vitreous fibril sample with increased exposure time of silver staining. The main ATTR fibril protein band in line 2 and 3 occurs at approximately 12 kDa. Two weak bands at 15 kDa (*) and 5 kDa (**) correspond to full length and shorter fragments of TTR, respectively. The results were reproduced in > three independent experiments. Source data are provided in the Source data file.

## Supplementary Figure 2

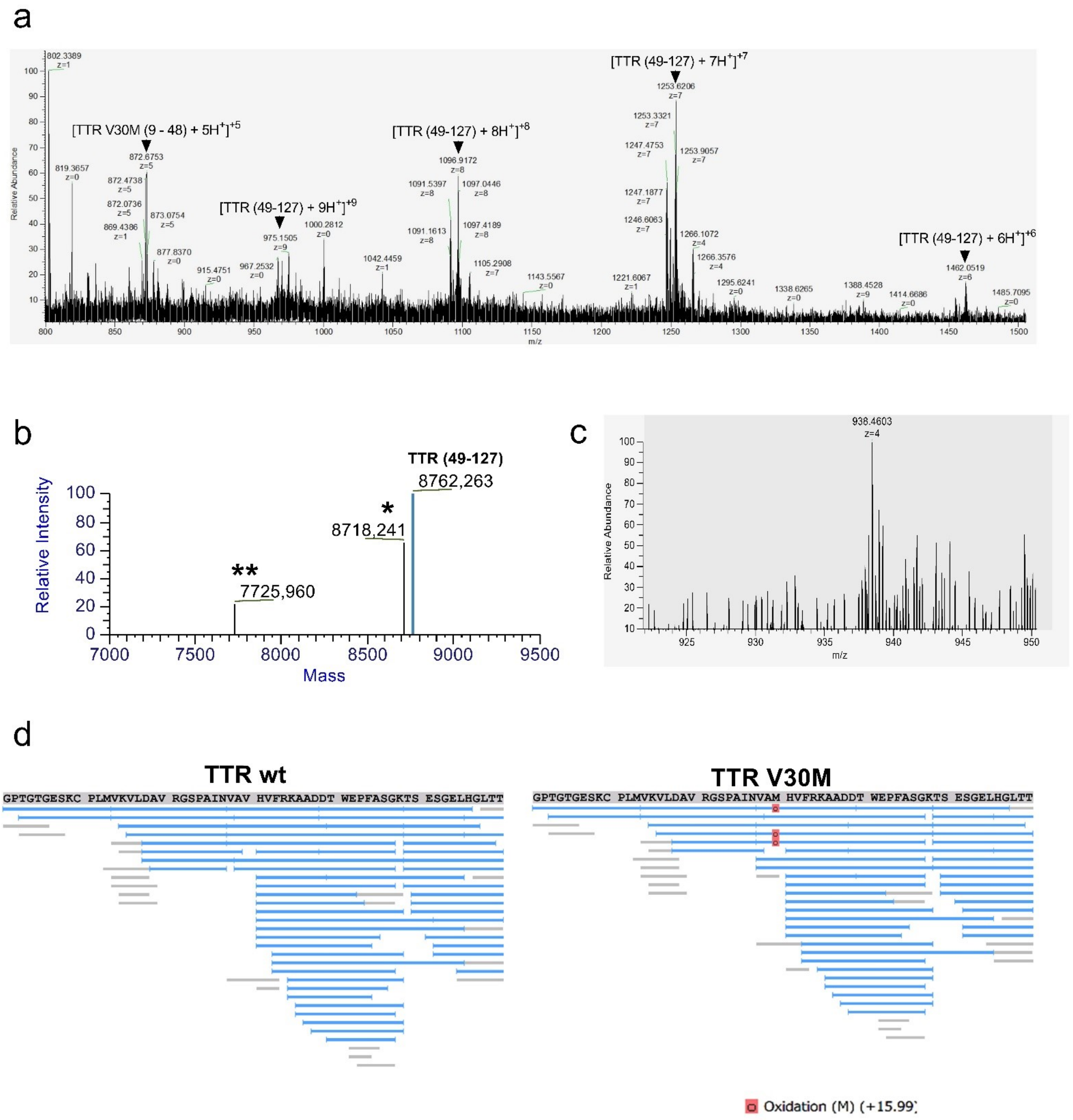


## Supplementary Figure 2

**Mass spectrometry analysis of vitreous ATTR fibrils**. a) Full mass spectra (spectral range 700-1,800 m/z) of the fibril solution. The spectra show presence of C- and N-terminal fragment ions with average masses of 4357,37 Da and 8767,34 Da corresponding to truncated TTR V30M (9-48) and TTR (49-127) respectively. b) Deconvoluted mass spectra (spectral range 7,000-9,500 Da shown) of mass spectra

in a. The spectra show the main product corresponding to truncated TTR (49-127) with monoisotopic mass of 8762,26 Da. The peak with monoisotopic mass of 8718,24 Da (*) suggests a chemically modified form of the fragment TTR (49-127). The mass shift of –44 Da could be possibly assigned as decarboxylation ($CO_2$, monoisotopic mass 43,98 Da) according to previous study[1] or it could correspond to fragment TTR (27-104)[2]. The fragment with monoisotopic mass of 7725,960 Da (**) could not be assigned within error (0.1 Da). c) Representative mass spectrum (spectral range m/z 920 to 950) showing an additional N-terminal fragment with sequence VKVLDAVRGSPAINVAVHVFRKAADDTWEPFASGK (residues 14-48, for z=4 theoretic m/z is 938.51) of TTR wt at an m/z value of 938.46. d) Pepsin-digested peptide coverage map of vitreous TTR wt and TTR V30M (residues 1-60). The analysis confirmed presence of both wt and variant TTR as well as possible oxidation of Met at position 30 (+15.99 Da, red).

**Supplementary Figure 3**

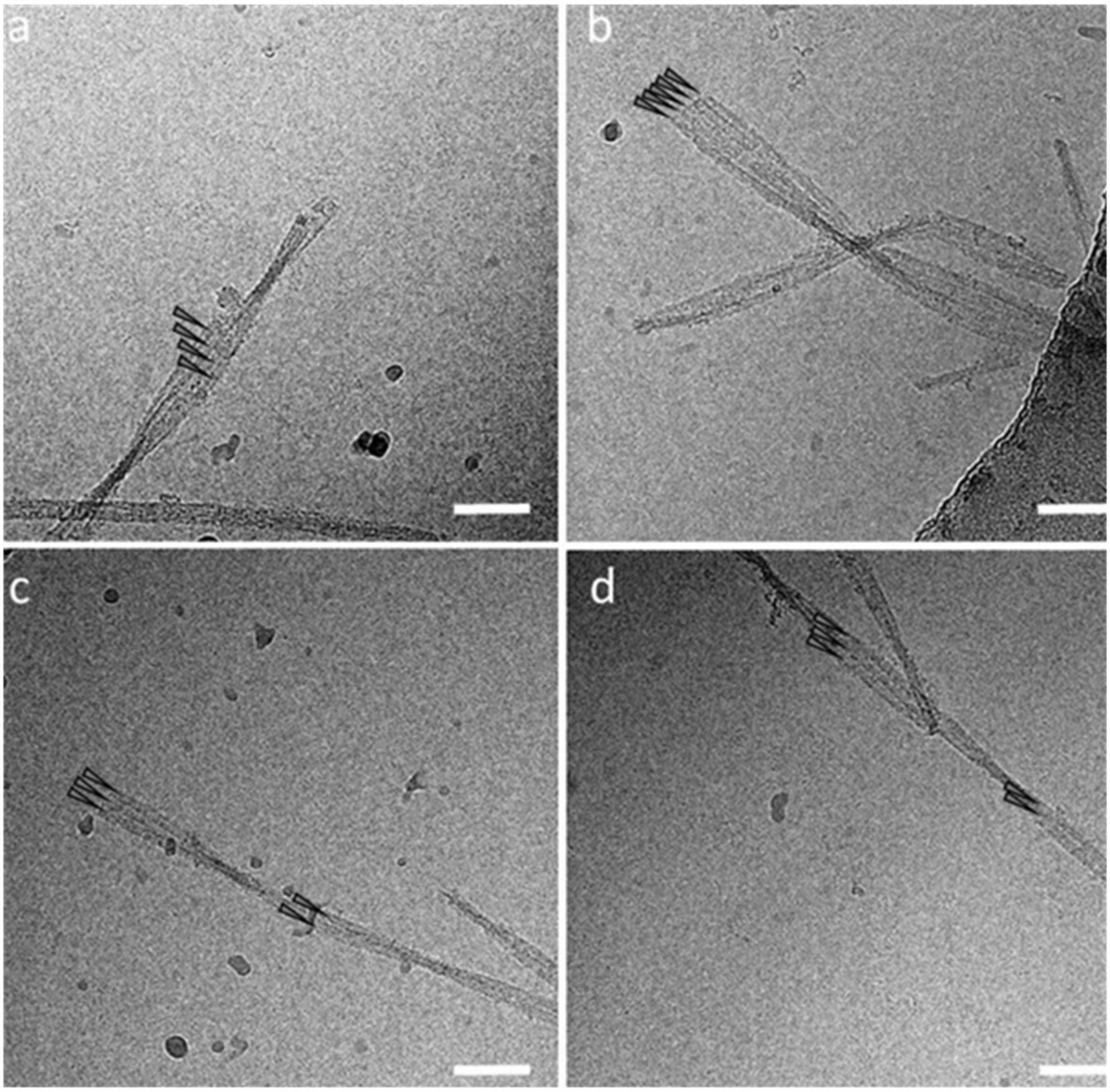


**Supplementary Figure 3**

**Polymorphism in vitreous sample.** Fibrils consisting of a) four and b) five protofilaments. Each protofilament is denoted with arrowheads. c-d) The coexistence of twisted-dimer and twisted-trimer in the same fibril. The scale bar is 50 nm. In total, 17,863 micrographs were analyzed from one fibril sample.

**Supplementary Figure 4**

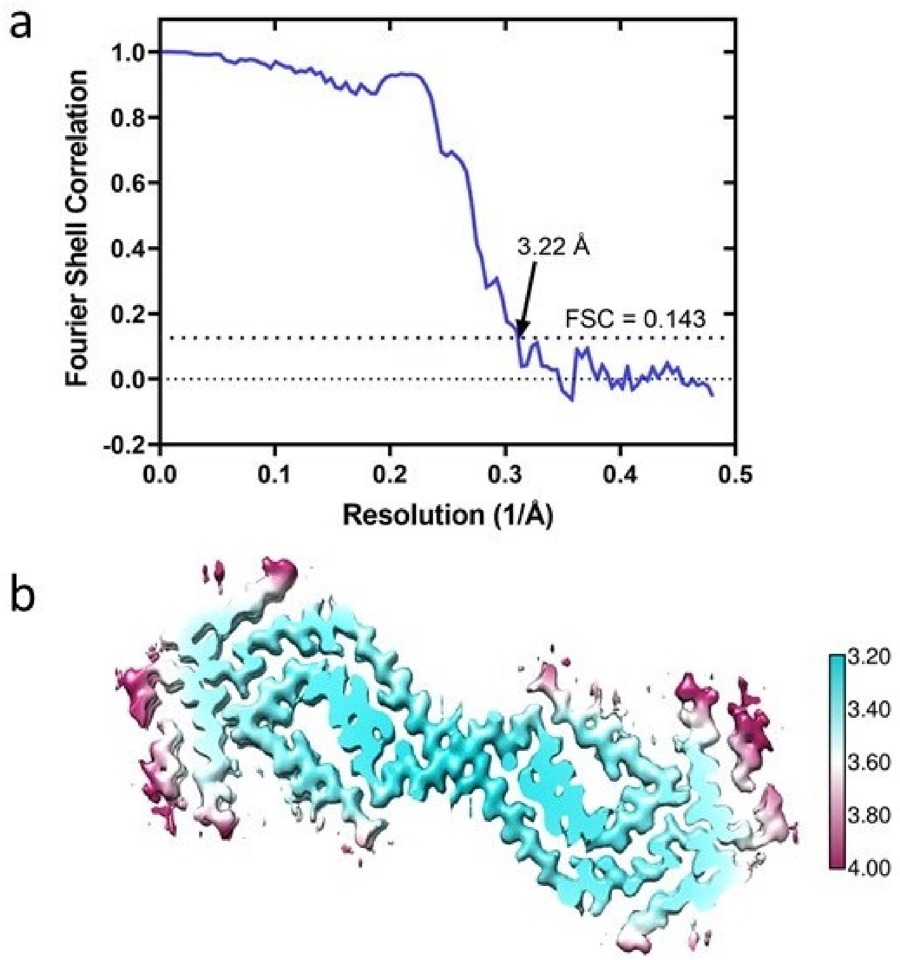


**Supplementary Figure 4**

**Cryo-EM map validation.** a) Gold-standard Fourier shell correlation (FSC) curve for estimating the resolution of the masked map. A cut-off value of 0.143 was used to calculate the final resolution. b) Local resolution estimation for the map. Source data for (a) are provided as a Source data file.

**Supplementary Figure 5**

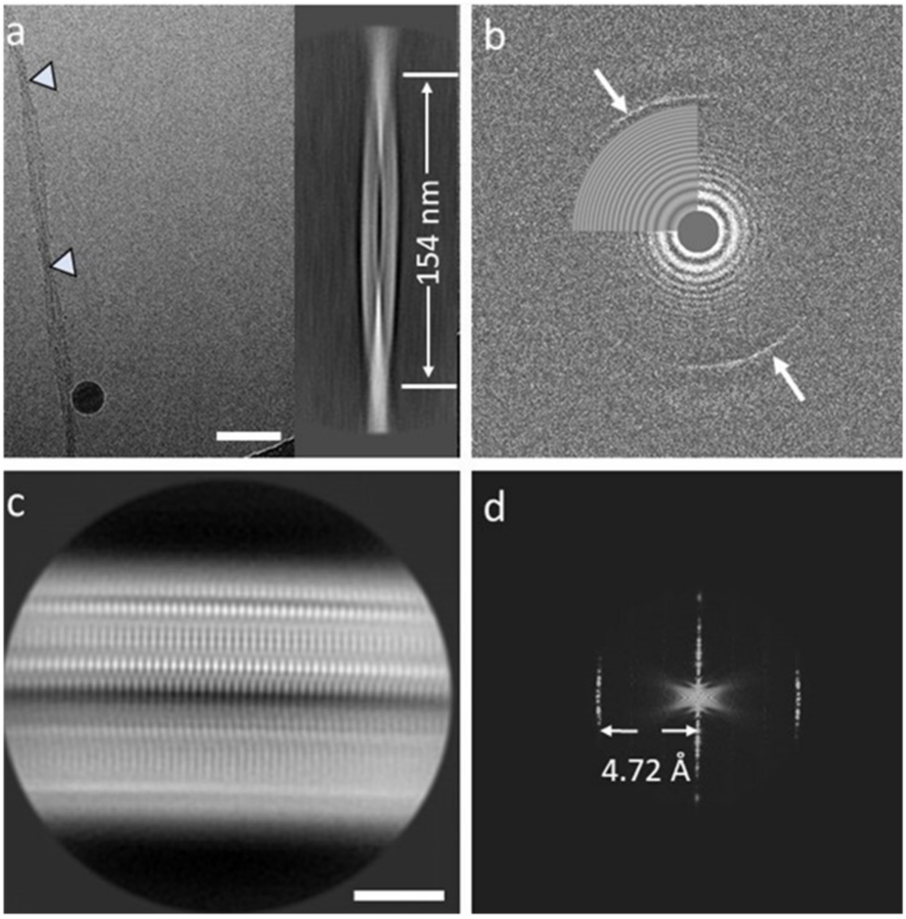


**Supplementary Figure 5**

**Estimation of helical parameters.** a) Cryo-EM image of a vitreous body ATTR fibril in vitreous ice. The distance between two adjacent crossovers is indicated with white arrowheads. The scale bar is 50 nm and in total, 17,863 micrographs were analyzed from one fibril sample. Inset: an average of fibril segments corresponding to half-pitch. b) The power spectrum of the micrograph presented in a. White arrows indicate layer lines corresponding to a helical rise of 1/4.72 Å. c) Reference-free 2D class average of vitreous body fibril showing periodicity, which is perpendicular to the long axis of the fibril. The scale bar is 50 Å. d) The power spectrum of the 2D class average illustrated in c. The layer line at 1/4.72 Å indicates the helical rise.

**Supplementary Figure 6**

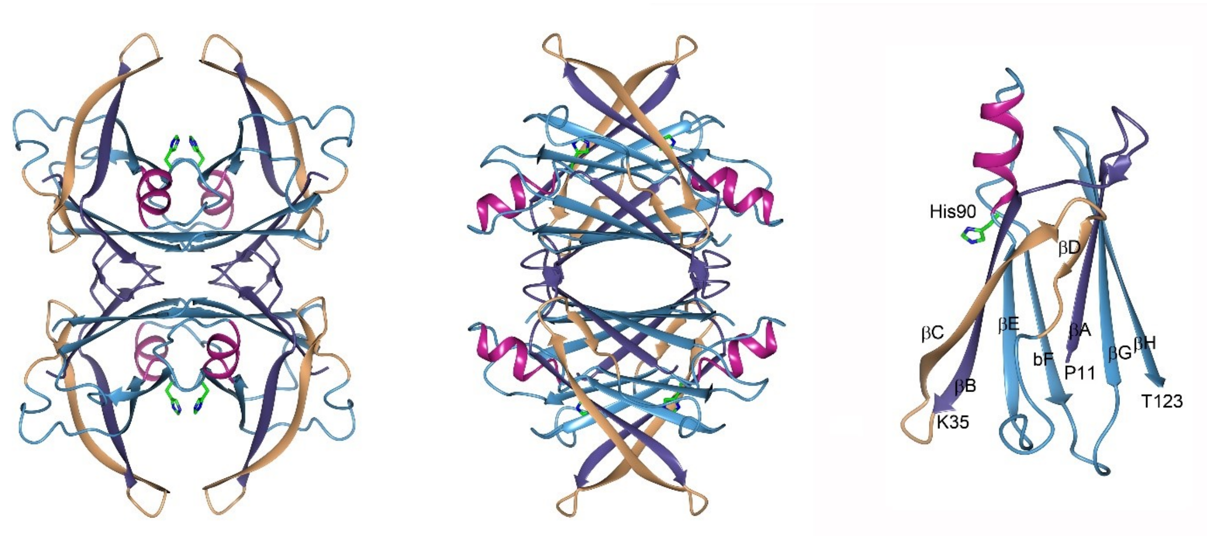


**Supplementary Figure 6**

**The native homo-tetrameric and monomeric structure of hTTR.** Two orientations (left, middle) of TTR tetramers followed by the monomer structure (right) (PDB-ID 1f41, the monomer is not drawn to scale with the tetramers). β-strands are named in the monomer structure. Color-coding is as follows: β-strands βA and βB in dark blue; residues Ala36–His56 including βC, βD and the CD-loop in orange; and βE, βF, βG and βH in light blue. The α-helix is highlighted in pink. The green sticks show the side chain of His90.

**Supplementary Figure 7**

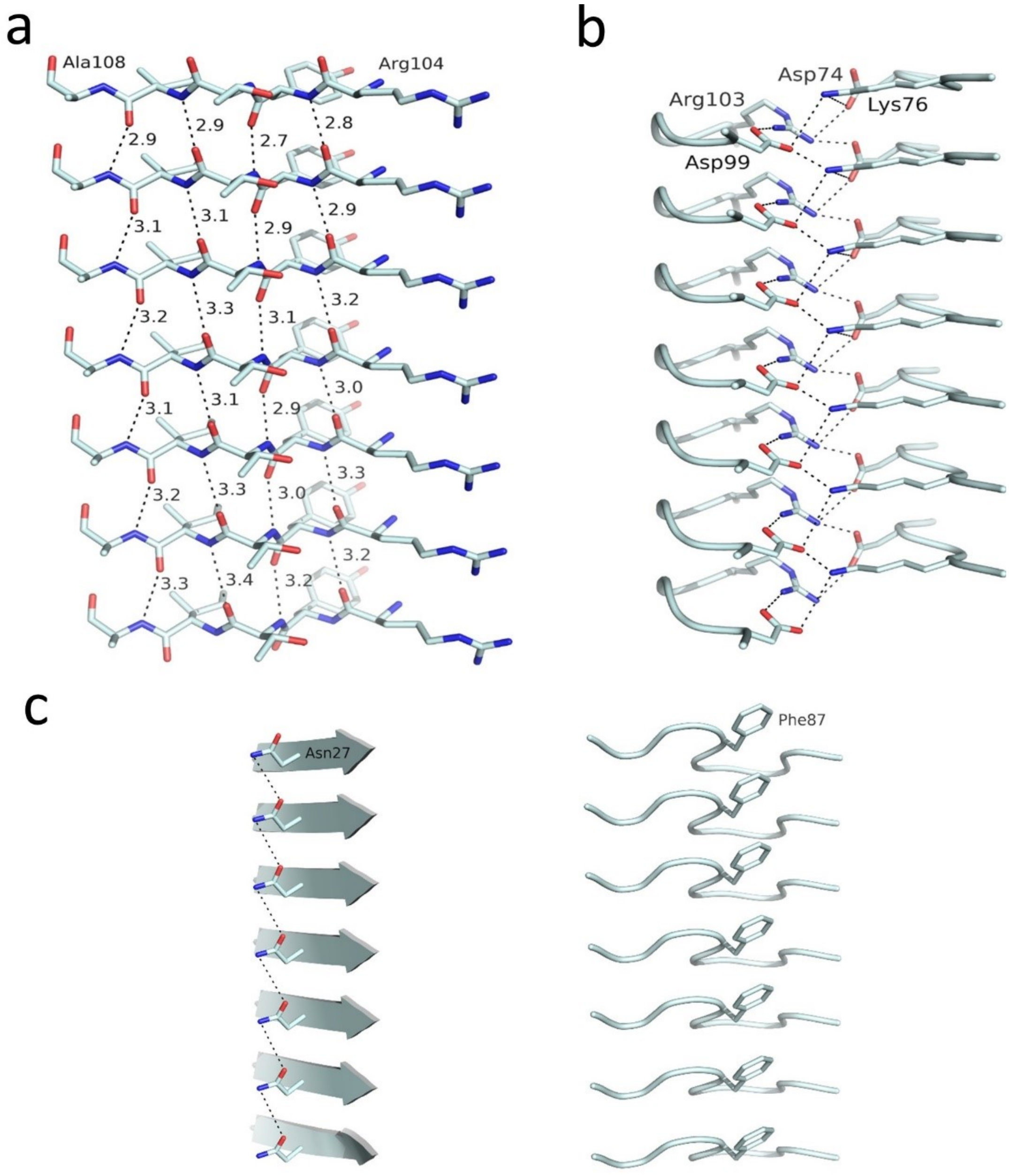


**Supplementary Figure 7**

**Structural features of fibril protein stacks packing into fibril.** a) Hydrogen bond network between main-chain atoms of parallel β-strands. b) Network of salt-bridges formed between the side chains of Asp74, Lys76, Asp99, and Arg103 positioned in different stacks. c) Examples of polar (Asn27) and aromatic (Phe87) stacking of residues along the main fibril axis.

**Supplementary Figure 8**

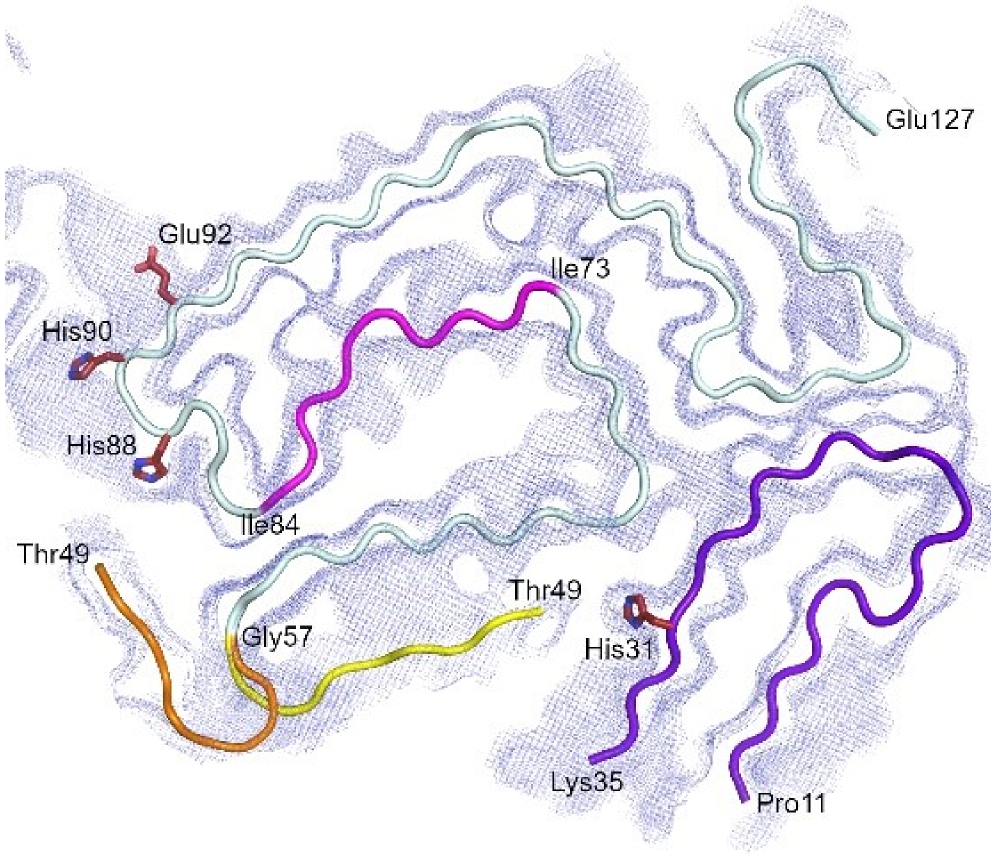


**Supplementary Figure 8**

**Tracing of C-terminal Thr49–Glu127 residue fragments in a protofilament of a vitreous body ATTR fibril.** The fragments are modelled in the light blue low-pass frequency map (filtered at 4.7 Å). The β-strands are not well separated at this low resolution, explaining why the density around the Cα-trace looks like hollow tubes. Color-coding of the fibril is as in Supplementary Figure 4. Two conformations of residues Thr49-His56 are shown in yellow and orange. To help guide the reader, some residues are shown as sticks.

**Supplementary Table 1**

**Cryo-EM data collection and image processing**

| ***Data collection*** | |
|---|---|
| Microscope | Titan Krios (Thermo Fisher Scientific) |
| Camera | K2 Summit (Gatan) |
| Acceleration voltage (kV) | 300 |
| Defocus range (µm) | -0.7 to -1.6 |
| Dose rate ($e^-$/$Å^2$/s) | 5.67 |
| Number of movie frames | 20 |
| Exposure time (s) | 5 |
| Total electron dose ($e^-$/$Å^2$) | 28.4 |
| Calibrated Pixel size (Å) | 1.041 |
| ***Helical Reconstruction*** | |
| Box size (pixel) | 220 |
| Inter box distance (Å) | 14.3 |
| Number of extracted segments | 130,212 |
| Number of segments after 2D classification | 128,269 |
| Number of segments after 3D classification | 27,778 |
| Helical rise (Å) | 4.719 |
| Helical twist (°) | -0.552 |

**Supplementary Table 2**

**Structural statistics of model building and refinement**

| ***Model composition*** | |
|---|---|
| Non-hydrogen atoms | 10052 |
| Protein residues | 1288 |
| Number of chains | 14 |
| Water/ligands | 0/0 |
| ***Model refinement*** | |
| Resolution (Å) | 3.2 |
| d FSC model (0/0.143/0.5) | 2.7/2.8/3.5 |
| Map CC (around atoms) | 0.78 |
| MolProbity score | 2.11 |
| R.m.s. deviations bonds (Å) | 0.009 |
| R.m.s. deviations angles (°) | 1.028 |
| B-factors ($Å^2$) (min/max/mean) | 23.3/92.9/50.0 |
| Clash score | 23.0 |
| Ramachandran (%) (outliers/allowed/favored) | 0.00/3.6/96.4 |
| Rotamer outliers (%) | 1.90 |
| Cβ outliers | 0.00 |
| CaBLAM outliers (%) | 5.70 |

## Supplementary References


1. Kingsbury, J.S. et al. Detailed Structural Analysis of Amyloidogenic Wild-Type Transthyretin Using a Novel Purification Strategy and Mass Spectrometry. *Analytical Chemistry* **79**, 1990-1998 (2007).
2. Schmidt, M. et al. Cryo-EM structure of a transthyretin-derived amyloid fibril from a patient with hereditary ATTR amyloidosis. *Nature Communications* **10**, 5008 (2019).
