# Supplementary material for: Structural basis for transthyretin amyloid formation in vitreous body of the eye
Source: Nat Commun. 2021 Dec 8;12:7141. doi: 10.1038/s41467-021-27481-4 (PMC8654999; doi:10.1038/s41467-021-27481-4)
Supplement: Supplementary file 2 — Reporting summary [file 41467_2021_27481_MOESM2_ESM.pdf]

nature portfolio

Corresponding author(s): Elisabeth Sauer-Eriksson

Last updated by author(s): November 12, 2021

Reporting Summary

Nature Portfolio wishes to improve the reproducibility of the work that we publish. This form provides structure for consistency and transparency in reporting. For further information on Nature Portfolio policies, see our Editorial Policies and the Editorial Policy Checklist.

Statistics

For all statistical analyses, confirm that the following items are present in the figure legend, table legend, main text, or Methods section.

n/a	Confirmed	
☐	☒	The exact sample size (n) for each experimental group/condition, given as a discrete number and unit of measurement
☐	☒	A statement on whether measurements were taken from distinct samples or whether the same sample was measured repeatedly
☒	☐	The statistical test(s) used AND whether they are one- or two-sided<br>*Only common tests should be described solely by name; describe more complex techniques in the Methods section.*
☒	☐	A description of all covariates tested
☒	☐	A description of any assumptions or corrections, such as tests of normality and adjustment for multiple comparisons
☐	☒	A full description of the statistical parameters including central tendency (e.g. means) or other basic estimates (e.g. regression coefficient) AND variation (e.g. standard deviation) or associated estimates of uncertainty (e.g. confidence intervals)
☒	☐	For null hypothesis testing, the test statistic (e.g. F, t, r) with confidence intervals, effect sizes, degrees of freedom and P value noted<br>*Give P values as exact values whenever suitable.*
☒	☐	For Bayesian analysis, information on the choice of priors and Markov chain Monte Carlo settings
☒	☐	For hierarchical and complex designs, identification of the appropriate level for tests and full reporting of outcomes
☒	☐	Estimates of effect sizes (e.g. Cohen's d, Pearson's r), indicating how they were calculated

Our web collection on statistics for biologists contains articles on many of the points above.

Software and code

Policy information about availability of computer code

Data collection	EPU 2.7.0
Data analysis	RELION 3.1, MotionCor2 1.3.0, CTFFIND 4.1.8, Coot 0.8.9.2, PHENIX 1.17.1, UCSF Chimera, Fiji (ImageJ) 1.52, BioPharma Finder 3.2, GraphPad Prism 9.2.0

For manuscripts utilizing custom algorithms or software that are central to the research but not yet described in published literature, software must be made available to editors and reviewers. We strongly encourage code deposition in a community repository (e.g. GitHub). See the Nature Portfolio guidelines for submitting code & software for further information.

Data

Policy information about availability of data

All manuscripts must include a data availability statement. This statement should provide the following information, where applicable:

- Accession codes, unique identifiers, or web links for publicly available datasets
- A description of any restrictions on data availability
- For clinical datasets or third party data, please ensure that the statement adheres to our policy

The reconstructed cryo-EM map of the twisted-dimer of ATTR Val30Met fibril from the vitreous body was deposited in the Electron Microscopy Data Bank with accession code EMD-12794 [https://www.ebi.ac.uk/pdbe/entry/emdb/EMD-12794]. The coordinates of the fitted atomic model were deposited in the Protein Data Bank under the accession code 7OB4 [https://doi.org/10.2210/pdb7OB4/pdb]. Other structural models used in this study are available in the Protein Data bank with entry codes: 6SDZ [https://doi.org/10.2210/pdb6sdz/pdb] (amyloid transthyretin from the heart); 6LNI [https://doi.org/10.2210/pdb6lni/pdb] (full-length human prion); 6DSO [https://doi.org/10.2210/pdb6dso/pdb] (murine amyloid A); and 6GK3 [https://doi.org/10.2210/pdb6gk3/pdb] (β2-microglobulin). The source data underlying Fig. 1b, Supplementary Figs. 1 and 4a are provided as a Source Data file with this paper. Other data that support the findings of this study are available

March 2021

from the corresponding authors upon reasonable request.

Field-specific reporting

Please select the one below that is the best fit for your research. If you are not sure, read the appropriate sections before making your selection.

☒ Life sciences ☐ Behavioural & social sciences ☐ Ecological, evolutionary & environmental sciences

For a reference copy of the document with all sections, see nature.com/documents/nr-reporting-summary-flat.pdf

Life sciences study design

All studies must disclose on these points even when the disclosure is negative.

Sample size	Vitreous material (ATTR fibrils) was collected by vitrectomy from a single patient
Data exclusions	130,212 segments were extracted from the raw data. 27,778 segments were used for reconstruction
Replication	Electron microscopy data based on a single human patient. In total 17,863 images were recorded and analysed during two separate data collections from one fibril sample. 27,778 segments were used for reconstruction
Randomization	Not relevant to study. Single case study
Blinding	Not relevant to study. Single case study

Reporting for specific materials, systems and methods

We require information from authors about some types of materials, experimental systems and methods used in many studies. Here, indicate whether each material, system or method listed is relevant to your study. If you are not sure if a list item applies to your research, read the appropriate section before selecting a response.

Materials & experimental systems

n/a	Involved in the study	
☒	☐	Antibodies
☒	☐	Eukaryotic cell lines
☒	☐	Palaeontology and archaeology
☒	☐	Animals and other organisms
☐	☒	Human research participants
☒	☐	Clinical data
☒	☐	Dual use research of concern

Methods

n/a	Involved in the study	
☒	☐	ChIP-seq
☒	☐	Flow cytometry
☒	☐	MRI-based neuroimaging

Human research participants

Policy information about studies involving human research participants

Population characteristics	Source of vitreous fibrils: Gender: male; age: 72; diagnosis: ATTRv amyloidosis; mutaion: Val30Met; residence: Sweden; type of fibril composition: Type A (fragmented).
Recruitment	Selected based on clinical findings. Vitreous material was collected by vitrectomy from the eye of a 72-year-old male Swedish carrier of the ATTR Val30Met variant who suffered from both polyneuropathy and cardiomyopathy. Previous biopsy analysis confirmed type A fibril composition and heterozygosity for Val30Met. This is a single case study.
Ethics oversight	Ethical approval was obtained from the ethical review board of Umeå University, reference number Dnr 2018-329-32M. Informed consent was obtained from the patient for the analysis of the amyloid deposits.

Note that full information on the approval of the study protocol must also be provided in the manuscript.
